# New records in vascular plants alien to Tenerife (Spain, Canary Islands)

**DOI:** 10.3897/BDJ.9.e62878

**Published:** 2021-04-26

**Authors:** Filip Verloove

**Affiliations:** 1 Meise Botanic Garden, Meise, Belgium Meise Botanic Garden Meise Belgium

**Keywords:** aliens, Canary Islands, ephemerophytes, naturalisation, range expansion, secondary distribution area, Spain, Tenerife

## Abstract

**Background:**

Recent fieldwork by the author in Tenerife, mostly between 2014 and 2019, yielded new records of alien vascular plants.

**New information:**

Fifteen taxa (*Acacia decurrens*, *A. mearnsii*, *Caesalpinia pulcherrima*, *Ensete ventricosum*, Eucalyptus camaldulensis subsp. arida, *E. cladocalyx*, *Euryops chrysanthemoides*, *Ficus elastica*, *Lippia alba*, *Pavonia sepioides*, *Pittosporum tobira*, Populus × canadensis, *Pyrostegia venusta*, *Ruellia dipteracanthus* and *Wigandia kunthii*) are reported for the first time from the Canary Islands. All were initially introduced on purpose, mostly as ornamentals, and recently started to escape from cultivation. Most of them are ephemerals or only locally established, but nearly all have the potential to naturalise in the future. Thirteen additional species are reported for the first time from Tenerife: *Atriplex nummularia*, *Bellis perennis*, *Chenopodium probstii*, *Coccoloba uvifera*, *Commelina benghalensis*, *Cuphea hyssopifolia*, *Eragrostis virescens*, *Lemna minuta*, *Malvastrum corchorifolium*, *Plerandra elegantissima*, *Psidium guajava*, *Thunbergia alata* and *Urochloa subquadripara*. Finally, some miscellaneous notes are provided on the presence of *Balanites aegyptiaca*, *Callistemon viminalis*, *Grevillea robusta* and *Passiflora caerulea* in Tenerife.

## Introduction

Despite the long tradition of studies on the flora of the Canary Islands, native as well as introduced, there is a constant and almost uninterrupted amount of new taxonomic and distributional data. Particularly the non-native flora is still imperfectly known and the number of new introductions, deliberate as well as accidental, still seems to increase, also in Tenerife.

This paper is a sequel to Verloove and Reyes-Betancort (2011) and Verloove (2017) and reports about newly-detected alien vascular plants found in Tenerife, mostly between 2014 and 2019. Recent new records dealing with particular taxonomic groups (mostly succulents from the Agavaceae and Cactaceae families) have already been published elsewhere (Verloove et al. 2017, Verloove et al. 2019). We here present new records for 32 taxa that are either new to the Canary Islands (or even Macaronesia as a whole), to the island of Tenerife or that are otherwise of interest.

Assessing the actual invasion status of these taxa is not straightforward and the eventual degree of naturalisation will only become clear over time. Many of the species presented in this account are currently considered ephemerals or are only locally more or less established. However, nearly all of them have the potential to naturalise or even become invasive, taking into account their reported weediness elsewhere in the world (compare with, for example, Randall 2017). The reproduction from seed or clonal reproduction of introduced species are a first, but essential step towards an incipient naturalisation process. The early detection of such events is crucial in invasion biology.

## Materials and methods

Records here presented are the result of several months of fieldwork in Tenerife conducted by the author, mainly between 2014 and 2019. Herbarium specimens were collected for most of the records and these are deposited in the herbaria BR (Meise Botanic Garden, Belgium) and/or ORT (Instituto Canario de Investigaciones Agrarias, Puerto de la Cruz, Tenerife) (herbarium acronyms according to Thiers 2020).

The actual presence or absence on the island of Tenerife of the non-native taxa here presented was each time compared with data provided by Hohenester and Welss (1993), Acebes Ginovés et al. (2010), the Euro+Med PlantBase (2020) and the Banco de Datos de Biodiversidad de Canarias (2020). For some recently-introduced species, several additional papers were checked as well.

The paper is divided into two parts. The first and major part deals with taxa that are either first reported from the Canary Islands as a whole or from Tenerife. Each entry includes the scientific name of the taxon (if useful, accompanied by one or more homo- or heterotypic synonyms), the family to which the taxon belongs (see below), kind of chorological novelty, enumeration of selected herbarium collections and/or personal observations, origin (primary as well as secondary distribution range) and its estimated degree of naturalisation in Tenerife.

If relevant, some additional information is also provided (nomenclatural or taxonomic comments etc.). In the second part, miscellaneous notes are presented on some other alien species that are of interest. For convenience, within each of these parts, all taxa are presented in alphabetical order.

Familial and generic classifications are in accordance with APG IV (Angiosperm Phylogeny Group - APG 2016). For the taxa treated herein, this means, for instance, that Chenopodiaceae are included in Amaranthaceae and Lemnaceae in Araceae.

## Data resources

Specimen information is deposited in the observation.org (https://observation.org/) online database that is also published through the Global Biodiversity Information Facility (GBIF; https://www.gbif.org/).

## Checklists

### New chorological data for the Canary Islands and Tenerife

#### Acacia decurrens

Willd., 1806

C7C90395-2CE8-5C50-A4D9-844AD9E1FE16

Acacia decurrens Sp. Pl. 4(2): 1072. 1806.

##### Distribution

TENERIFE: Tegueste, Camino Urb. Las Rosetas close to TF-154 road, shrubland, a single (?) shrub amongst numerous *A. mearnsii*, 18.01.2019, *F. Verloove* 13446 (BR). https://observation.org/observation/204629654/

##### Notes

*Acacia decurrens* is endemic to New South Wales in Australia. However, its natural distribution is uncertain as a result of frequent naturalisation, caused by widespread plantings as an ornamental and in forestry plantations. It is now commonly naturalised in other parts of Australia, but also in, for example, South Africa and California. In some areas, it is considered to be a very troublesome weed (Miller et al. 2011, Sunardi and Titiek 2017).

From the Canary Islands, it had not been reported yet (Acebes Ginovés et al. 2010). A single shrub was found amidst a shrubland that mostly consisted of *Acacia mearnsii* in Tegueste, Tenerife. It may have been deliberately introduced there a long time ago.

This species is most readily distinguished from the other two Australian bipinnate-leaved acacias that are commonly grown in the Canary Islands (i.e. *A. dealbata* and *A. mearnsii*) by its branchlets that are acutely angled by obvious winged ridges and its longer, narrowly linear leaflets (Maslin et al. 2019).

#### Acacia mearnsii

De Wild., 1925

92B3C395-0DFF-52F4-AA61-F2438D7C58F0

Acacia mearnsii Pl. Bequaert. 3(1): 62–63. 1925.

##### Distribution

TENERIFE: Tegueste, Camino Urb. Las Rosetas close to TF-154 road, shrubland, 18.01.2017, *F. Verloove* 13419 (BR). https://observation.org/observation/204629675/

##### Notes

This species is a native of south-eastern Australia, but introduced into many countries for utilisation purposes (mostly as an ornamental shrub). It easily reproduces and, like other acacias, is often considered to be an invasive species (Miller et al. 2011, Luque et al. 2014).

**Figure 1.**
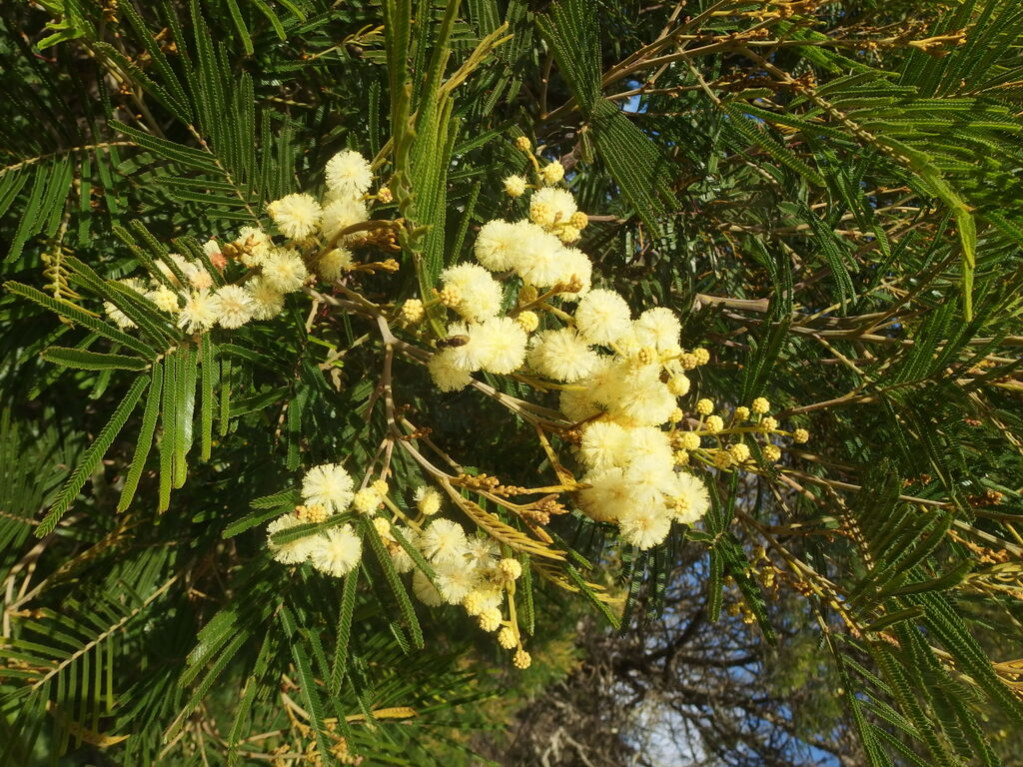
*Acacia mearnsii*, Tegueste, January 2019. Compared with *A. dealbata*, this species has a green, shiny foliage and cream flowers.

In Tenerife, *Acacia dealbata* is a naturalised shrub, although some records doubtlessly refer to *A. mearnsii* instead. The latter superficially resembles *A. dealbata*. It is recognised by its green and shiny foliage (vs. foliage bluish-grey with lustrous leaflets), cream flowers (vs. bright yellow flowers) (Fig. 1) and pods softly appressed grey-pubescent to velutinous (vs. pods glabrous). Furthermore, in *A. dealbata*, there is only a single gland at the base of the pinnae, whereas in *A. mearnsii* on at least some leaves, multiple glands are present between the pinnae (Maslin et al. 2019).

In Tegueste, *Acacia mearnsii* was probably initially planted a long time ago and now survives. In the same place, a single individual was also observed of *A. decurrens* (see earlier).

#### Atriplex nummularia

Lindl., 1848

4B4AD93B-15B9-5A4A-A2FE-670A605B692B

Atriplex nummularia J. Exped. Trop. Australia 64. 1848.

##### Distribution

TENERIFE: San Cristóbal de La Laguna, Bajamar, TF-13 road N of the village, roadside, +/- 10 individuals, 07.11.2014, *F. Verloove* 11242 (BR). https://observation.org/observation/204634934/

##### Notes

This Australian shrub is sometimes introduced in arid, harsh areas (e.g. Middle East, North Africa), mostly as an ornamental or as a windbreak, for erosion control, forage etc. It occasionally reproduces from seed, naturalises and is sometimes considered to be an undesirable weed. For example, it is a top ten prominent invader in the Nama-Karoo and Succulent Karoo biomes in South Africa (Henderson 2007). In the Canary Islands, it was recently reported from Fuerteventura and Gran Canaria (Verloove 2013, Verloove and Guiggi 2013). A small naturalised population with ca. 10 individuals has been known from Bajamar in Tenerife for many years.

*Atriplex nummularia* is much reminiscent of *A. halimus* L., a species that naturally occurs in the Canary Islands. However, at least part of these populations undoubtedly refers to introduced races. For instance, a well-known expansive population from Las Chafiras (Barone 2003), also in Tenerife, consists of diploids, whereas native populations are tetraploids (comm. A. Reyes-Betancort). The latter possibly correspond with var. schweinfurthii Boiss. (compare with Walker et al. 2005), a variety that occurs in arid zones with milder winters. *A. nummularia*, in turn, is an octoploid (Sampson and Byrne 2012). It usually is dioecious and *A. halimus* monoecious, although exceptions to this rule occur.

#### Bellis perennis

L., 1753

71E1F50A-1C9D-579A-83FE-0ACE0E17D8AD

Bellis perennis Sp. Pl. 2: 886. 1753.

##### Distribution

TENERIFE: San Cristóbal de La Laguna, Cruz del Carmen, Anaga Mountains, parking *mirador*, bare, trodden soil, in several small subpopulations, 10.01.2017, *F. Verloove* 12708 (BR). https://observation.org/observation/204635431/

##### Notes

This Eurasian weed is widely naturalised in temperate areas across the world; see, for instance, Brouillet (2006). According to Acebes Ginovés et al. (2010), only *B. annua* L. has been recorded so far in the Canary Islands (solely from Tenerife). However, these records may at least partly refer to *B. perennis*. For instance, from the natural protected area ‘Anaga Rural Park’ – where we have found *B. perennis* – *B. annua* was cited by Expósito et al. (2018). *B. perennis* is a perennial, stoloniferous species with thick fleshy roots and all leaves in a basal rosette. *B. annua*, in turn, is annual and has leafy stems.

*Bellis perennis* was recently recorded for the first time in the Canary Islands by Otto and Verloove (2016). The species was found as a weed in irrigated lawns in La Palma. In identical circumstances, it was also observed for the first time in Gran Canaria (Verloove et al. 2020).

#### Caesalpinia pulcherrima

(L.) Sw., 1791

1CAD60FE-FF83-5805-8AB3-DB35F175B739

Caesalpinia pulcherrima Observ. Bot. 166. 1791.

##### Distribution

TENERIFE: Adeje, Playa de Las Américas, barranco at Av. Eugenio Dominguez Alfonso, dry riverbed close to the sea, a single individual, self-sown, ca. 150 cm tall (cultivated nearby), 09.01.2017, *F. Verloove* 12714 (BR). https://observation.org/observation/204635636/

##### Notes

This species is widely grown as an ornamental shrub. In fact, its exact origin is unknown due to widespread cultivation. It is a prickly shrub or small tree with bipinnate, showy red and yellow flowers with very long stamens (60 mm or more long) with yellow or bright red filaments and relatively large petals (more than 25 mm long).

*Caesalpinia pulcherrima* is fast-growing and easily reproduces from seed. Therefore, it is naturalised in many regions where it was introduced in the past and it is increasingly considered an invasive weed (for instance, in parts of Australia, Ecuador, the Philippines and Cuba).

**Figure 2.**
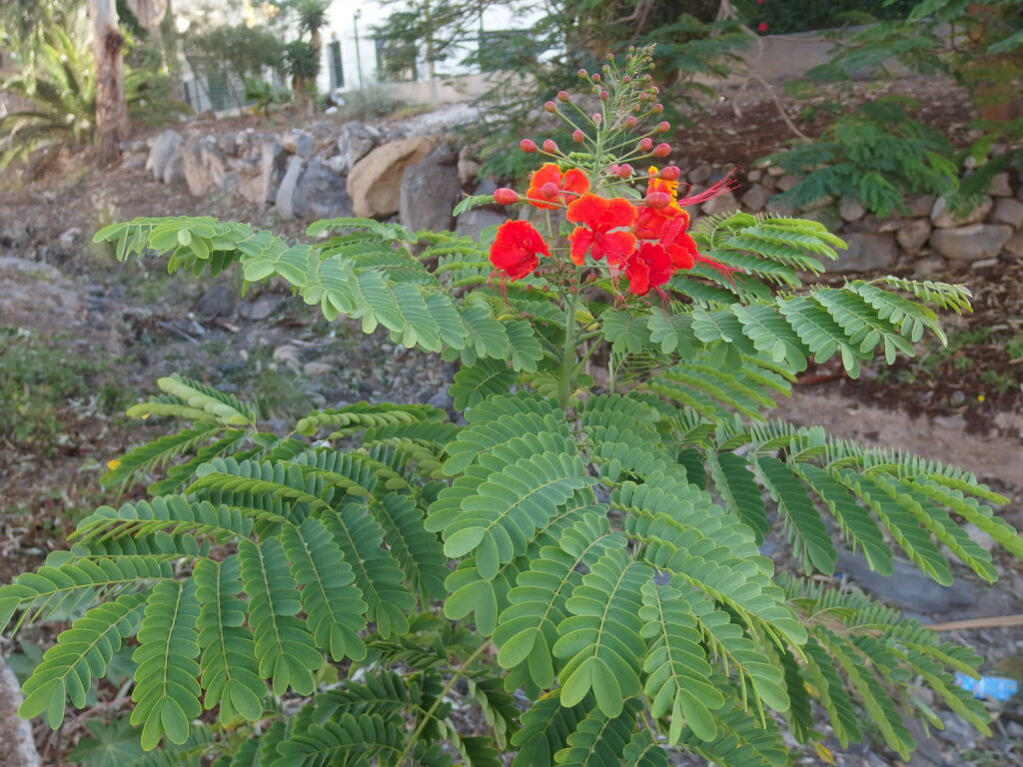
*Caesalpinia pulcherrima*, Adeje, January 2017.

In January 2017, a single self-sown flowering individual was observed in a dried-out water channel close to the sea in Playa de Las Américas in Tenerife (Fig. 2).

#### Chenopodium probstii

Aellen, 1928

B20A3A06-04DA-5B81-B380-3CAD08E5410B

Chenopodium probstii Mitt. Naturf. Ges. Solothurn 20(8): 56. 1928.

##### Distribution

TENERIFE: Los Silos, Erjos, charcas de Erjos, dried out pond, very common, but only locally, 18.11.2016, *F. Verloove* 12679 (BR). https://observation.org/observation/205254266/

##### Notes

This weed of uncertain origin (possibly North America) doubtlessly has been overlooked. It was recently reported from several localities in La Palma, for the first time in the Canary Islands, where it is considered to be naturalised and in expansion (Otto and Verloove 2016). It differs from *C. album* by leaves that are large and leathery and with a distinct purple or orange pigmentation.

It is here reported for the first time from Tenerife. In and near some dried out ponds in Erjos, it was seen in abundance in 2016. It probably also occurs elsewhere.

#### Coccoloba uvifera

(L.) L., 1759

41D9BC84-285E-5ABF-BB2A-A1689C207F03

Coccoloba uvifera Syst. Nat. (ed. 10)2: 1007. 1759.

##### Distribution

TENERIFE: Santiago del Teide, Puerto de Santiago (Los Gigantes), Paseo Marítimo, sea cliff below coastal path, a single, self-sown shrub, mass-planted nearby, 10.12.2019, *F. Verloove* 13722 (BR). https://observation.org/observation/205254392/

##### Notes

*Coccoloba uvifera* is native to coastal beaches throughout tropical America and the Caribbean. It is wind- and salt-tolerant and very often planted in ornamental plantations in warm-temperate and subtropical areas across the world, especially near the coast. It is very commonly grown in the Canary Islands as well. Fruits (sea grapes) are produced abundantly and relatively easily germinate. However, since ornamental plantings usually are found in urban habitats, regeneration is rarely observed. When planted in more natural suitable habitats, *C. uvifera* readily naturalises and is sometimes considered an environmental weed.

**Figure 3.**
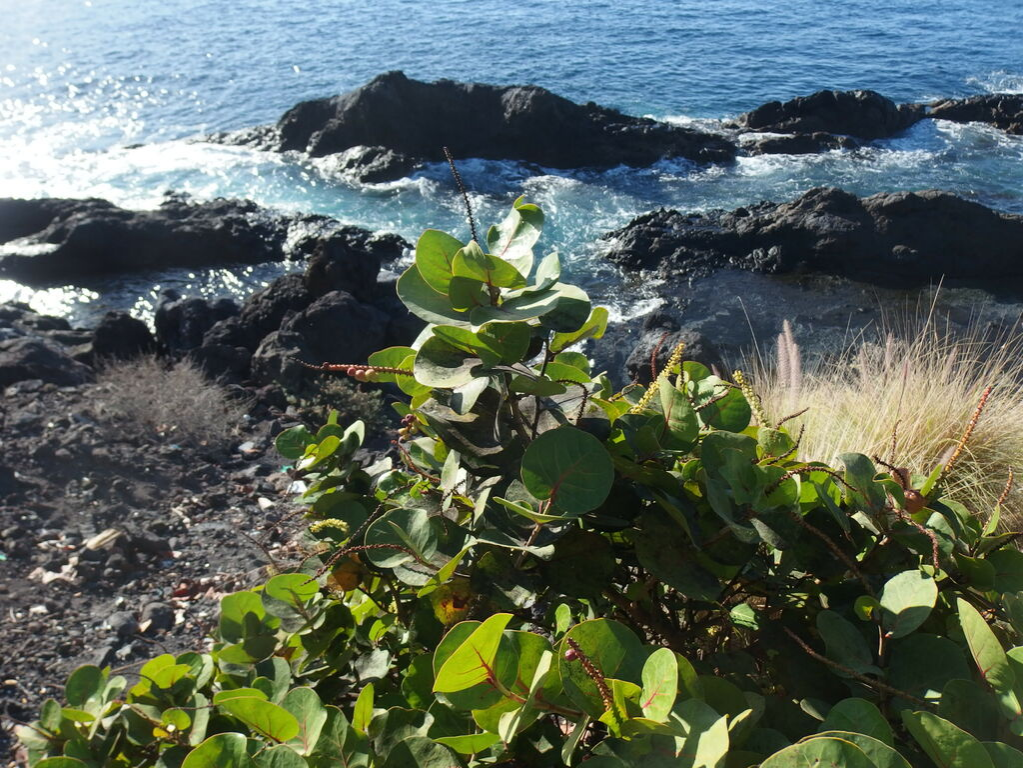
*Coccoloba uvifera*, flowering and fruiting in Santiago del Teide (Puerto de Santiago), December 2019.

Despite being very commonly planted, the species had not been recorded so far as an escape in the Canary Islands until recently, when it was observed to copiously regenerate in two localities in La Palma (Otto and Verloove 2016). In December 2019, a single self-sown shrub (flowering and fruiting) was observed growing on a sea cliff below a coastal path in Puerto de Santiago (Fig. 3). It obviously escaped from a nearby plantation. For a number of reasons, a future local naturalisation in the Canary Islands is not unlikely: there is a climatic match, numerous suitable habitats and plenty of seed sources.

#### Commelina benghalensis

L., 1753

55F06B6D-5A82-5B36-B663-EFB7C829377C

Commelina benghalensis Sp. Pl. 1: 41. 1753.

##### Distribution

TENERIFE: Arona, Palm-Mar, Paseo Avutarda, wall of manhole, persistent since 2015, 18.12.2018, *F. Verloove* 13426 (BR). https://observation.org/observation/205254429/

##### Notes

*Commelina benghalensis* is a weed from the Old World subtropics. In the Canary Islands, it is only known from Gran Canaria (Acebes Ginovés et al. 2010), although most (if not all) records probably date back to the 19^th^ century (e.g. Buch 1833).

Since 2015, it has been known from a single locality in Tenerife. In Palm-Mar, it persists on the wall of a manhole. Its presence was regularly confirmed since then.

This species differs from the common and invasive *Commelina* species in the Canary Islands (*C. diffusa*) by the spathe margins that are fused near base. This characteristic is shared with *C. latifolia* Hochst. ex A. Rich., an African species that recently naturalised in La Palma (Otto and Verloove 2016). It can be distinguished from the latter by the typical pubescence that consists of reddish hairs and all petals, including the minute lower one, being blue.

#### Cuphea hyssopifolia

Kunth, 1823 [1824]

E44697E4-72B1-5939-9A61-418F59C9B58C

Cuphea hyssopifolia Nov. Gen. Sp. (quarto ed.) 6: 199–200. 1823 [1824]

##### Distribution

TENERIFE: Santa Úrsula, La Quinta, Calle El Escoban, cracks in pavement, three escaped individuals (not seen planted nearby), 10.12.2018, *F. Verloove* 13416 (BR). https://observation.org/observation/205254459/

##### Notes

This tiny ornamental shrub is native to Mexico, Guatemala and Honduras, but widely cultivated elsewhere in the subtropics. It easily self-seeds and sometimes naturalises where introduced, for instance, in Hawaii and New Zealand (Gardner and de Lange 1996, Gardner 1998). In the Canary Islands, it was previously reported from La Palma (Otto and Verloove 2016).

In December 2018, few plants were noticed in cracks of concrete in a residential area in La Quinta.

#### Ensete ventricosum

(Welw.) Cheesman, 1947 [1948]

25BC9BA2-CCD1-5535-9F0F-4C0CF815CBA0

Ensete ventricosum Kew Bulletin 1947(2): 101. 1947 [1948]

##### Distribution

TENERIFE: Santa Cruz de Tenerife, Las Casas de la Cumbre, 10.01.2017, *F. Verloove* s.c.https://observation.org/observation/205254987/

##### Notes

**Figure 4.**
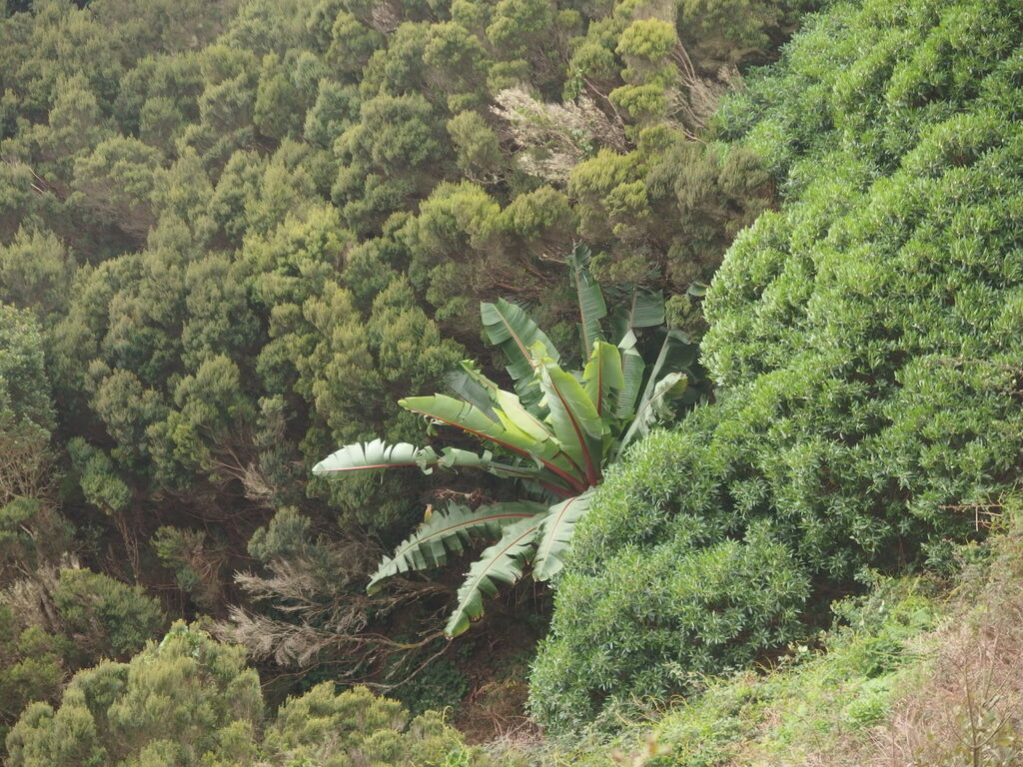
*Ensete ventricosum*, Santa Cruz de Tenerife (Las Casas de la Cumbre), January 2017.

This species, known as Ethiopian banana, is cultivated as staple food in parts of East Africa. In addition, it is grown as an ornamental, also in the Canary Islands. In the Anaga Mountains in Tenerife, it is locally planted in roadsides near urbanisations. In 2017, a single individual was observed in an impenetrable thicket, composed of native low shrubs (Fig. 4). It is unknown whether this plant refers to a former plantation or germinated from seed.

In New Zealand, *Ensete ventricosum* is known to have naturalised in comparable climatological circumstances (Gardner and de Lange 1996).

#### Eragrostis virescens

J. Presl, 1830

69DC7C18-F4C3-53FD-B118-4ABD6598D686

Eragrostis virescens Reliq. Haenk. 1(4–5): 276. 1830.
Eragrostis virescens Syn.: Eragrostis mexicana (Hornem.) Link subsp. virescens (J. Presl) S.D. Koch & Sánchez Vega, Phytologia 58(6): 380. 1985.

##### Distribution

TENERIFE: Santa Úrsula, La Quinta, alongside dirt tracks, rather numerous in two localities, 11.12.2018, *F. Verloove* 13410 (BR, ORT). https://observation.org/observation/205255016/

##### Notes

This New World weed is increasingly naturalised in Europe (Martini and Scholz 1998). In the Canary Islands, it was first reported from La Palma (Otto et al. 2008). Since 2012, it is also known from two localities in Gran Canaria (Marrero Rodríguez 2019). In December 2018, *Eragrostis virescens* was observed in relative abundance along dirt tracks in Santa Úrsula, for the first time in Tenerife.

This species looks fully naturalised. A further spread in similar habitats in the area can be expected.

#### Eucalyptus camaldulensis arida

Brooker & McDonald, 2009

3442DF8E-0ABB-5670-9556-B9C7A324ED3D

Eucalyptus camaldulensis arida Austral. Syst. Bot. 22(4): 273. 2009.

##### Distribution

TENERIFE: Santiago del Teide, centre of the village, steep slope of abandoned quarry, planted and freely escaping, 14.01.2019, *F. Verloove* 13447 (BR). https://observation.org/observation/205256471/

##### Notes

*Eucalyptus camaldulensis* is commonly planted in the Canary Islands and relatively easily self-seeds. The plants that are usually seen have opercula which are strongly beaked; they belong to subsp. camaldulensis. In Gran Canaria, subsp. arida has also been recorded, although only as a planted tree. It is often found in mixed plantations with subsp. camaldulensis (Marrero Rodríguez 2016). It is distinguished by the mature buds that have an obtuse to rounded, not beaked, operculum.

In Santiago del Teide in Tenerife, these two subspecies are mass-planted in and on the verge of an abandoned quarry. Both are reproducing from seed.

#### Eucalyptus cladocalyx

F. Muell., 1852 [1853]

BE8AD6E5-750F-5C84-8E1E-FFC22C4D3586

Eucalyptus cladocalyx F. Muell., Linnaea 25(4): 388–389. 1852 [1853].

##### Distribution

TENERIFE: Santa Úrsula, La Quinta, rough ground, mass-planted and escaping, 15.01.2017, *F. Verloove* 12753 (BR); Santa Úrsula, La Quinta, Eucalyptus woodland, planted and escaping, 08.12.2018, *F. Verloove* 13413 (BR). https://observation.org/observation/205256523/; https://observation.org/observation/205256567/

##### Notes

This species is native to parts of south-eastern South Australia. It is frequently introduced elsewhere, either as an ornamental tree, a windbreak or for timber plantations. It easily escapes from many of these plantings and is now regarded as an environmental weed in other parts of Australia. It has also naturalised overseas in southern Africa, California (U.S.A.) and Hawaii.

In the Canary Islands, this tree is known to be cultivated as was shown in an extensive overview of the genus for Gran Canaria (Marrero Rodríguez 2016). However, sexual reproduction was not yet observed for that species. In Santa Úrsula (La Quinta) in Tenerife, *Eucalyptus cladocalyx* is mass-planted in several localities. Saplings have been recorded on several occasions.

#### Euryops chrysanthemoides

(DC.) Nordenstam, 1968.

3B2E24BB-C69F-5442-B80D-ECB1A1CD2820

Euryops chrysanthemoides (DC.) Nordenstam, Opera Bot. 20: 365–370, f. 62C–G, 63C. 1968.

##### Distribution

TENERIFE: Icod de los Vinos, La Vega, TF-373 road near km 4, on top of stone (rock) wall along the road, four clumps, possibly a relic of former cultivation (?), 14.12.2019, *F. Verloove* 13724 (BR). https://observation.org/observation/205285538/

##### Notes

*Euryops chrysanthemoides* is native to the Cape Province of South Africa. It is frequently cultivated as an ornamental and easily escapes; as a result, it is also found as a weed of roadsides, disturbed areas and urban open spaces. It is sometimes considered to be an invasive species, especially in parts of Africa where it is not native (Henderson 2002, Witt and Luke 2017). It is also weedy in, for instance, Australia, New Zealand and Hawaii.

In La Vega in Tenerife, scattered bushes are growing alongside the road, on top of a rock wall. This species had not been recorded before in the Canary Islands.

#### Ficus elastica

Roxb. ex Hornem., 1819.

58D57B0A-026C-5D9C-9C93-88DA727D45A3

Ficus elastica Suppl. Hort. bot. hafn. 7. 1819.

##### Distribution

TENERIFE: Arona, Chayofa, barranco de la Arena S of the village, dry riverbed, a single large clone, 16.03.2016, *F. Verloove* 12457 (BR). https://observation.org/observation/205285557/

##### Notes

**Figure 5.**
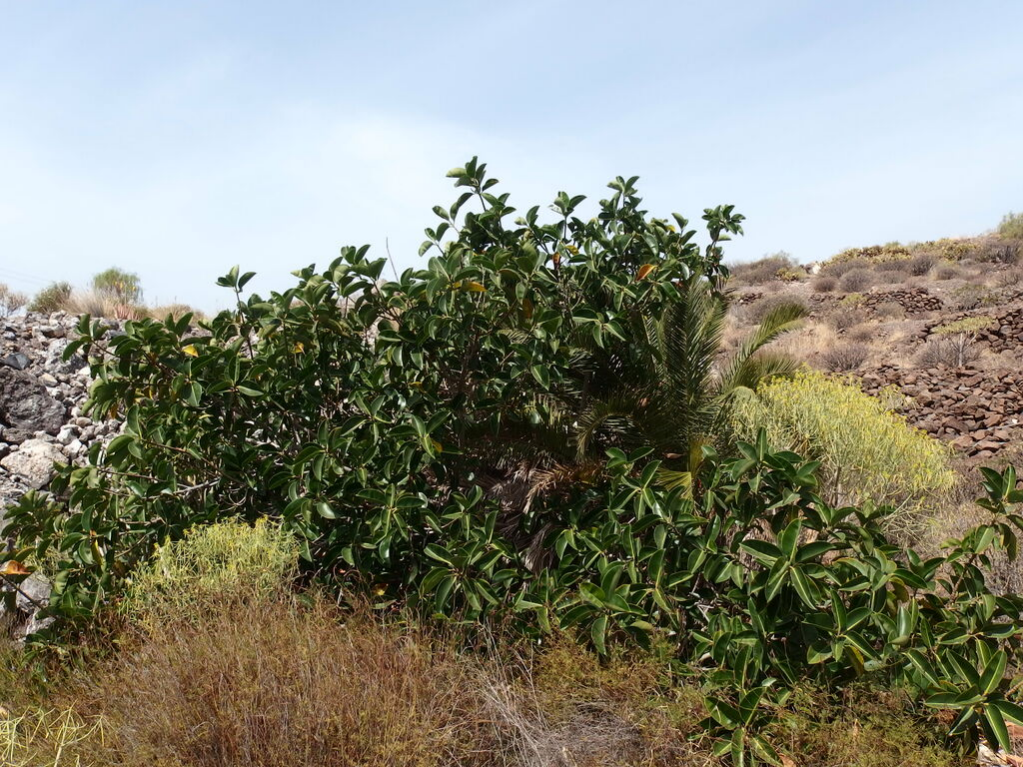
*Ficus elastica*, Arona (Chayofa), March 2016.

*Ficus elastica* is native in south-eastern Asia, but one of the most frequently cultivated species of the genus. It has large, glabrous and leathery leaves and reddish stipules, 10-15 cm long that cover the terminal buds. In Chayofa, a single large clone is growing in a dried-out ravine (Fig. 5), probably from washed-up rhizomes or garden debris.

Several species of *Ficus* have been recorded in the wild in the Canary Islands, especially *F. microcarpa* and (to a lesser extent) *F. lyrata* and *F. rubiginosa* and these are increasing lately. *F. elastica* is reported here for the first time. Despite being commonly grown, it is only rarely observed as an escape, for instance, in Florida in the United States (Wunderlin 1997). In some areas, it has naturalised or even become invasive, although this entirely depends on whether its specialist pollinator wasp has also been introduced to the area (Starr et al. 2003).

#### Lemna minuta

Kunth, 1815 [1816].

958492D4-4DF3-501D-9A0C-B24E3F4EE647

Lemna minuta Nov. Gen. Spec. (quarto ed.) 1: 372. 1815 [1816].

##### Distribution

TENERIFE: Santa Úrsula, La Quinta, seepage area, 11.12.2018, *F. Verloove* s.c. https://observation.org/observation/205285824/

##### Notes

This American duckweed is a recent newcomer in the flora of the Canary Islands. It was first reported from several localities in Gran Canaria (Verloove 2013, Salas-Pascual and Quintana Vega 2016), subsequently also from La Palma (Otto and Verloove 2020). It is a naturalised weed that also occurs in natural habitats. As it strongly resembles *L. minor* L., it is undoubtedly overlooked. *L. minuta* is differentiated from the latter, based on the smaller fronds (even the largest are less than 3 mm long) with a single, slightly raised vein.

In La Quinta, a small population was discovered in a seepage zone in 2018. It should be looked for elsewhere in the northern part of the Island.

*Lemna minuta* is a well-known transformer species throughout the invaded range (Paolacci et al. 2018).

#### Lippia alba

(Mill.) N.E. Br. ex Britton & P. Wilson, 1925.

FC2705AD-578D-5101-BDF6-82985BC3A59F

Lippia alba Bot. Porto Rico 6(1): 141. 1925.

##### Distribution

TENERIFE: Guía de Isora, Playa de San Juan, SE of the village, shallow, dry barranco, a single shrub, relic of former plantation (?), 14.01.2019, *F. Verloove* 13438 (BR); Santa Cruz de Tenerife, San Andrés, Barranco de San Andrés, dry gravelly riverbed, two young shrubs, self-sown, 22.12.2019, *F. Verloove* 13738 (BR).https://observation.org/observation/205285935/; https://observation.org/observation/205286074/

##### Notes

*Lippia alba* is native to southern Texas (U.S.A.), Mexico, the Caribbean, Central America and South America. It is widely cultivated as an ornamental or for its aromatic foliage. In places where it was formerly introduced, it relatively easily naturalises, for instance, in Australia (Munir 1993). In Europe, it was recently reported for the first time from a single locality in Portugal (Verloove and Alves 2016).

**Figure 6.**
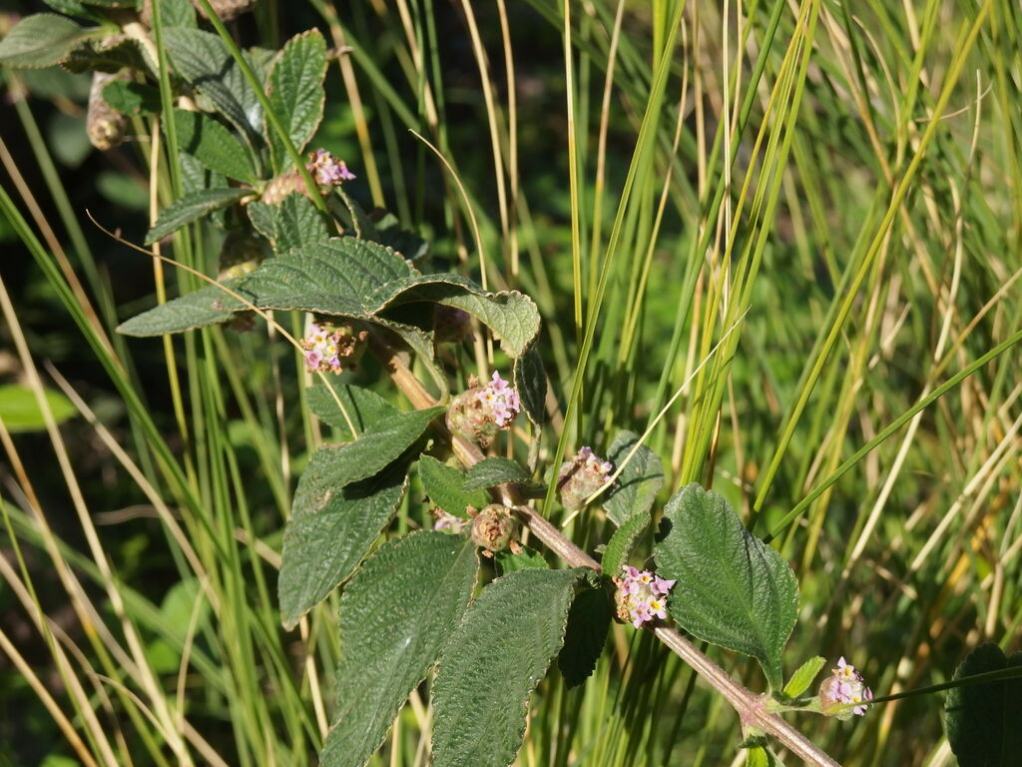
*Lippia alba*, Santa Cruz de Tenerife (San Andrés), December 2019.

In 2019, this species was recorded in two localities in Tenerife. In Playa de San Juan, a single shrub grows in a very shallow, dried-out ravine. It may be a mere relic of former cultivation there. In a second locality, in San Andrés, however, two young, obviously self-sown shrubs were found growing on the gravel of the dry riverbed (Fig. 6).

#### Malvastrum corchorifolium

(Desr.) Britton ex Small, 1913.

93673297-6263-50B2-8AAD-DA64A2200D61

Malvastrum corchorifolium Fl. Miami 119. 1913.

##### Distribution

TENERIFE: Granadilla de Abona, El Médano, beach, barranco de los Calderones, from sewage sludge, a single individual, 14.11.2016, *F. Verloove* 12680 (BR, LPA). https://observation.org/observation/205286307/

##### Notes

This species is native to Mexico, the West Indies, Central America and Florida in the U.S.A. It is weedy and often encountered elsewhere in the subtropics, for instance, in Africa (Ghana), but also in the Canary Islands. In La Palma, it was reported for the first time by Santos Guerra et al. (2013), whereas Kunkel (1968) already reported it from San Lorenzo in Gran Canaria a long time ago. The latter occurrence was apparently overlooked by Acebes Ginovés et al. (2010).

The *Malvastrum* weed, usually seen in the Canary Islands, for instance, in Gran Canaria where it is relatively frequent and much increasing lately, is *M. coromandelianum* (L.) Garcke. None of these two species has been reported before from Tenerife. In El Médano, a single individual of *M. corchorifolium* was observed in November 2016. The plant grew on the beach in a cumulation area of sewage sludge, along with tomatoes and other plants that germinated from the sewage water. It was no longer seen in the intervening years.

In general appearance *Malvastrum corchorifolium* resembles *M. coromandelianum* a lot (it probably is a hybrid of it). However, its mature carpels are muticous or have at most a blunt apical protuberance less than 0.2 mm long, whereas in the latter, mericarps are clearly aristate.

#### Pavonia sepioides

Fryxell & Krapov., 1999.

CC987561-7F2C-5C2E-B3D3-64B283658FAB

Pavonia sepioides Fl. Neotrop., Monogr. 76: 221–222, f. 73. 1999.

##### Distribution

TENERIFE: Puerto de la Cruz, Calle Camelia, foot of fence of Hotel Botanico, ca. 10-15 individuals, 15.12.2019, *F. Verloove* 13733 (BR). https://observation.org/observation/205286366/

##### Notes

*Pavonia sepioides* naturally occurs in Bolivia, Colombia, Ecuador and Venezuela. It was only recently described (Fryxell 1999) as a segregate of *P. sepium* A. St.-Hil., a more southern species with its main distribution in Brazil and neighbouring territories. These two species, as well as *P. spinifex* (L.) Cav., belong to Pavonia sect. Urenoideae A. St.-Hil. and are not easily distinguished. They are sometimes grown as ornamentals, especially in the subtropics and tropics, although probably not frequently so (none is mentioned, for instance, by Bird 2011, but see Huxley (1999). Some are also found as weeds (Holm et al. 1979, Randall 2017). *P. spinifex* is probably most widely grown (e.g. Sánchez de Lorenzo Cáceres 2000). It has large flowers 40-70 mm across and ovate leaves with cordate bases (Fryxell 1999), unlike the plants recently found in Tenerife. These have narrowly lanceolate leaves and much smaller flowers. They correspond with the species formerly named *P. sepium*. Fryxell (1999) segregated plants from northern South America as *P. sepioides*. With the exception of the presence of a few hair tufts in the axils of the nerves of the lower leaf surface in *P. sepium*, these two species largely overlap in all further character states. These hair tufts are not seen in the Tenerife plant material and, for this reason, they are here ascribed to *P. sepioides*. It probably is not a coincidence that escaped and locally-naturalised plants in tropical East Africa (Uganda) were also attributed to that species (Verdcourt and Mwachala 2009). '*P. sepium*' has reportedly been known as a garden ornamental in Tenerife (Reyes Betancort and Pérez-de-Paz 2000). From Portugal, '*P. sepium*' was recently reported by Almeida de and Freitas (2012). The species escaped from a Botanic Garden in Lisbon and exhibits invasive behaviour (Forte et al. 2011).

**Figure 7.**
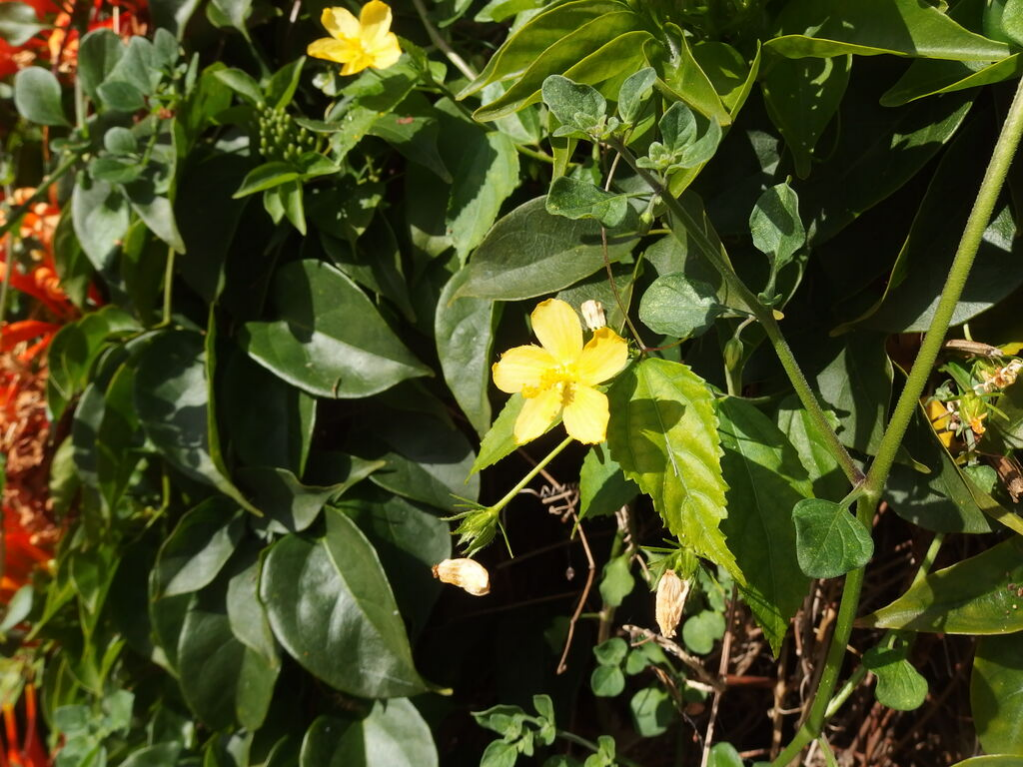
*Pavonia sepioides*, Puerto de la Cruz, December 2019.

The provenance of the plants observed in Tenerife is unknown. They grow relatively near to the Botanic Garden and on the verge of a hotel garden (foot of fence, covered in *Pyrostegia venusta*) and may have escaped from one of these (Fig. 7). On the other hand, the fruits are long-spined and retrorsely barbed and therefore easily attach to clothing and fur. As a result, species of section Urenoideae have been introduced widely to foreign regions (see also Fryxell and Hill 2015 for *P. spinifex*).

#### Pittosporum tobira

(Thunb.) W.T. Aiton, 1811.

A81BBA1E-FBC0-576C-800F-E308E1182F36

Pittosporum tobira Hortus Kew. (2^nd^ ed.) 2: 27. 1811.

##### Distribution

TENERIFE: Tegueste, El Socorro, TF-154 road, as epiphyte on *Phoenix*, rather numerous individuals in several trees on both sides of the road, 18.01.2019, *F. Verloove* 13445 (BR). https://observation.org/observation/205286418/

##### Notes

A native of East Asia, this shrub is frequently cultivated as an ornamental in warm-temperate areas across the world. Its seeds are embedded in a resinous pulp which probably explains why the species is frequently dispersed by berry-eating birds. As a result, *Pittosporum tobira* is regularly found as an epiphyte on palm trees, just like species of the genera *Ficus* or *Schefflera*.

In El Socorro in Tenerife, bird-sown shrubs of this species have been observed for several years on *Phoenix* trunks.

From the same genus, the Australian shrub *Pittosporum undulatum* Vent. is an invasive species in Gran Canaria and Tenerife (Acebes Ginovés et al. 2010), especially in the evergreen laurel forest. However, it is also sometimes observed as an epiphyte on *Phoenix*, for instance, in Puerto de la Cruz.

#### Plerandra elegantissima

(Veitch ex Mast.) Lowry, G.M. Plunkett & Frodin, 2013.

DB8BFBF7-066E-5060-9505-EC11C6C1325C

Plerandra elegantissima Brittonia 65: 49. 2013.
Plerandra elegantissima Syn.: *Schefflera elegantissima* (Veitch ex Mast.) Lowry & Frodin, Baileya 23: 9. 1989; *Dizygotheca elegantissima* (Veitch ex Mast.) R. Vig. & Guillaumin, Notul. Syst. (Paris) 2: 258. 1912; *Aralia elegantissima* Veitch ex Mast., Gard. Chron. 1873: 782. 1873.

##### Distribution

TENERIFE: Puerto de la Cruz, in front of Hotel Masaru, in planter, a sapling ca. 150 cm tall, self-sown, 14.12.2019, *F. Verloove* 13727 (BR). https://observation.org/observation/205286440/

##### Notes

This species was initially described as a species of *Aralia* L., then transferred to *Schefflera* J.R. Forst. & G. Forst. However, molecular studies (Lowry II et al. 2013) have demonstrated that *Schefflera*, the largest genus of the Araliaceae family, is grossly polyphyletic. The species, belonging to the Melanesian clade, are preferably accommodated in a separate genus, *Plerandra* A. Gray. It counts 32 species that are restricted to Melanesia (Fiji, New Caledonia, New Guinea, the Solomon Islands and Vanuatu). A few species are commonly cultivated as ornamentals, especially *P. elegantissima*.

*Plerandra elegantissima* is endemic to New Caledonia, but widely cultivated for its decorative juvenile foliage that is palmately divided, formed by 7-12 almost linear leaflets with grossly and irregularly dentate margins. Like other species of *Schefflera* s.l., fruits are drupaceous and thus consumed by berry-eating birds. As a result, ornamental species of this genus are easily dispersed; they are particularly frequent as epiphytes on palm trees, also in the Canary Islands, where *S. actinophylla* (Endl.) Harms and *S. arboricola* (Hayata) Merr. are increasingly recorded (e.g. Verloove and Reyes-Betancort 2011, Verloove 2017, Otto and Verloove 2018). Up to now, *P. elegantissima* had only been recorded from La Palma in the Canary Islands (Otto and Verloove 2016). It was observed to freely reproduce from seed in ornamental gardens and public greens. In Tenerife, it was seen in comparable circumstances in December 2019.

*Plerandra elegantissima* is an emerging invasive weed in some parts of the world, for instance, in South Africa (several online references).

#### Populus × canadensis

Moench, 1785

FFD189B6-7848-5AC1-BAE3-446EF95D3DA0

Populus × canadensis Verz. Ausländ. Bäume 81. 1785.

##### Distribution

TENERIFE: Puerto de la Cruz, barranco San Felipe, dry riverbed, a single individual (self-sown), 16.01.2017, *F. Verloove* 12752 (BR); Puerto de la Cruz, barranco San Felipe, dry gravelly riverbed, a single self-sown tree ca. 400 cm tall (also planted nearby), 17.12.2019, *F. Verloove* 12752 (BR). https://observation.org/observation/205286560/

##### Notes

Populus × canadensis is a hybrid of *P. nigra* L. and *P. deltoides* Marshall. It occurs naturally in areas where both species grow sympatrically, but is also very widely grown as an ornamental tree. It rather grows in temperate climates and is thus less frequently seen in the Canary Islands.

In Puerto de la Cruz in Tenerife, trees planted on the verge of a ravine in an urban environment have reproduced from seed. This hybrid had not been reported before from the Canary Islands (Acebes Ginovés et al. 2010).

#### Psidium guajava

L., 1753

8FFCCBEC-3845-5E47-9C3B-4E9855B72E1D

Psidium guajava Sp. Pl. 1: 470. 1753.

##### Distribution

TENERIFE: Santa Cruz de Tenerife, barranco Santos at Calle de Diego Crosa, dry riverbed, scattered individuals, 13.11.2016, *F. Verloove* 12684 (BR). https://observation.org/observation/205286613/

##### Notes

*Psidium guajava*, a native of Central and South America, is widely cultivated in tropical and subtropical regions around the world for its edible fruits (guava). It easily escapes wherever introduced. In the Canary Islands, it was first reported from various localities in La Palma (Santos Guerra and Reyes-Betancort 2014), shortly afterwards also from Gran Canaria (Verloove 2017). It is mostly found in dried-out riverbeds where it germinates from wastewater. In such circumstances, it was also recently found for the first time in Tenerife.

#### Pyrostegia venusta

(Ker Gawler) Miers, 1863.

10DF55C7-E410-522D-9B1A-F7AD45C82D04

Pyrostegia venusta Proc. Roy. Hort. Soc. London 3: 188. 1863.

##### Distribution

TENERIFE: Santa Úrsula, La Quinta, barranco de la Plaza, shrubland adjacent to barranco, at Hotel La Quinta, escape (also elsewhere in the area), 15.01.2017, *F. Verloove* 12729 (BR). https://observation.org/observation/205286641/

##### Notes

*Pyrostegia venusta* is native to Brazil, but widely cultivated in the tropics and subtropics as an ornamental vine. It is very expansive and readily colonises vast surfaces. Although the seed-set is rarely observed outside the native range, the species is increasingly considered an unwanted, invasive environmental weed that quickly spreads as a result of clonal growth. It is now classified as an invasive weed in many areas, for instance, in Florida in the U.S.A. (Hutchinson 2005).

**Figure 8.**
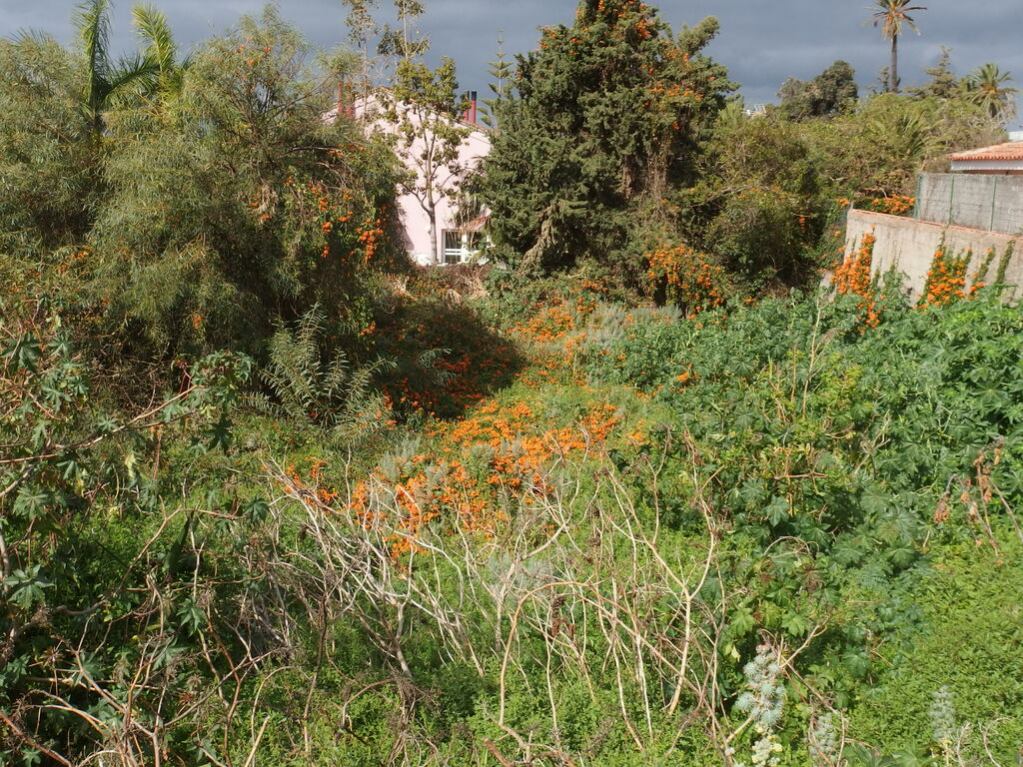
*Pyrostegia venusta*, Santa Úrsula (La Quinta), January 2017.

*Pyrostegia venusta* grows in several places in La Quinta in Tenerife (Fig. 8). It is found in vacant lots in residential areas and on the verge of a ravine. It probably arose from discarded garden waste. It had not been recorded before in the Canary Islands. Since it is very commonly grown as an ornamental there, it will doubtlessly increase in the near future and may well establish permanent colonies.

#### Ruellia dipteracanthus

(Nees) Hemsl., 1882.

44EE8B96-4B3C-565D-8143-29DD0E563501

Ruellia dipteracanthus Biol. Cent.-Amer., Bot. 2(12): 504. 1882.
Ruellia dipteracanthus Syn.: *R. squarrosa* (Fenzl) Cufod., Baileya 17: 40 1970.

##### Distribution

TENERIFE: Santa Cruz de Tenerife, Igueste de San Andrés, Carretera de Igueste de San Andrés N of the Barranco de San Andrés, foot of steep rocks, small population, 23.12.2019, *F. Verloove* 13742 (BR). https://observation.org/observation/205286653/

##### Notes

*Ruellia dipteracanthus* is a native of Mexico, but regularly grown as a garden ornamental in warm-temperate and subtropical regions in the world, often under several other names, including *R. squarrosa* and *R. bremeri*. It differs from similar species, based largely on its low, sprawling stature and smaller leaves. This species is increasingly naturalised outside its native range, for instance, in the southern U.S.A. (Keith et al. 2017) or in Australia where it is a minor or emerging environmental weed in south-eastern Queensland and a potential environmental weed or "sleeper weed" in other parts of Australia. It is also considered an invasive species in Japan (Mito and Uesugi 2004).

**Figure 9.**
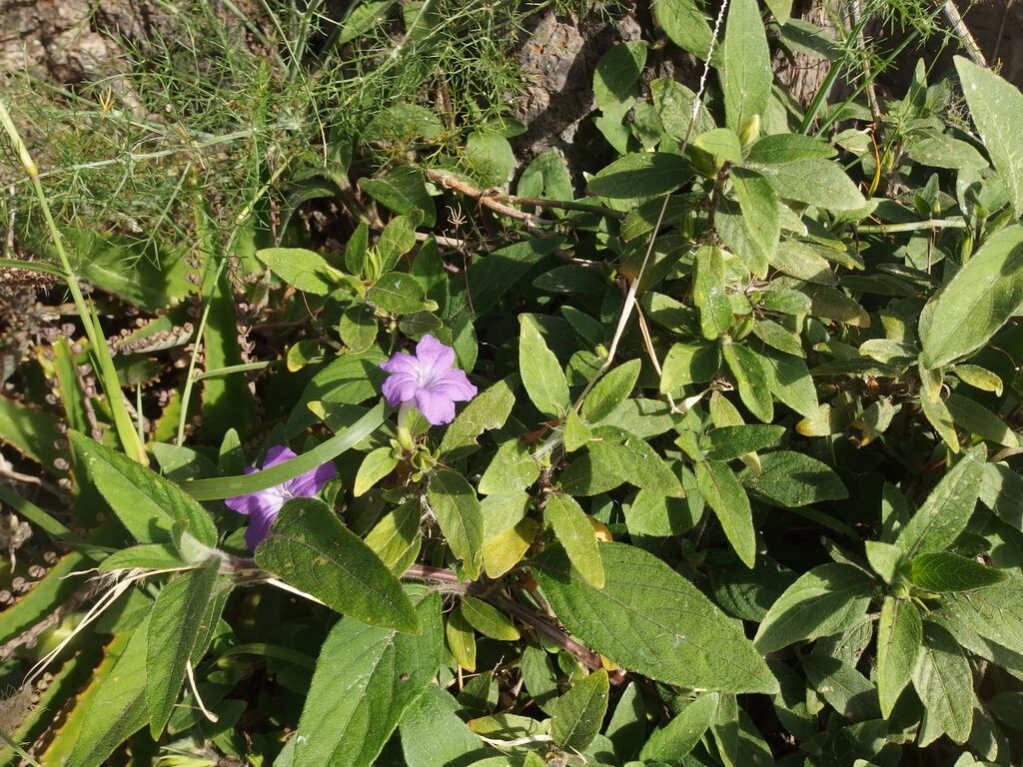
*Ruellia dipteracanthus*, Santa Cruz de Tenerife (Igueste de San Andrés), December 2019.

In December 2019, a small population (or a single large clone?) of this species was found sprawling on and at the foot of a damp, steep rock alongside the road (Fig. 9). The plants most likely escaped from a nearby garden although it was not seen planted in the surroundings.

Its identification was not straightforward, also because it is not included in garden flora accounts, such as Percy (2011) and Huxley (1999). *Ruellia dipteracanthus* is a low, creeping plant with ovate to narrowly-ovate leaves that are hairy. Its lavender tubular flowers have five small narrow sepals (10-16 mm long) and fruits are glabrous.

This and several other species of *Ruellia* are often invasive environmental weeds in the subtropics. These species seed profusely and also reproduce vegetatively via creeping underground stems and stem segments.

#### Thunbergia alata

Bojer ex Sims, 1825.

061E4081-B94F-5701-BE51-CD6A5416ACE5

Thunbergia alata Bot. Mag. 52: pl. 2591. 1825.

##### Distribution

TENERIFE: San Cristóbal de La Laguna, Tejina, Barranco de Las Cuevas at Calle Jose Rodríguez Amador, slope of ravine, a dense stand, but only very locally, 09.01.2017, *F. Verloove* 12712 (BR). https://observation.org/observation/205286762/

##### Notes

This East African species is commonly grown as an ornamental vine and easily escapes from cultivation. It is widely naturalised in warm-temperate and subtropical areas across the world to such an extent that it is often considered to be an environmental weed. It was recently reported for the first time in the Canary Islands from several localities in La Palma, where it is naturalised now (Otto and Verloove 2018).

**Figure 10.**
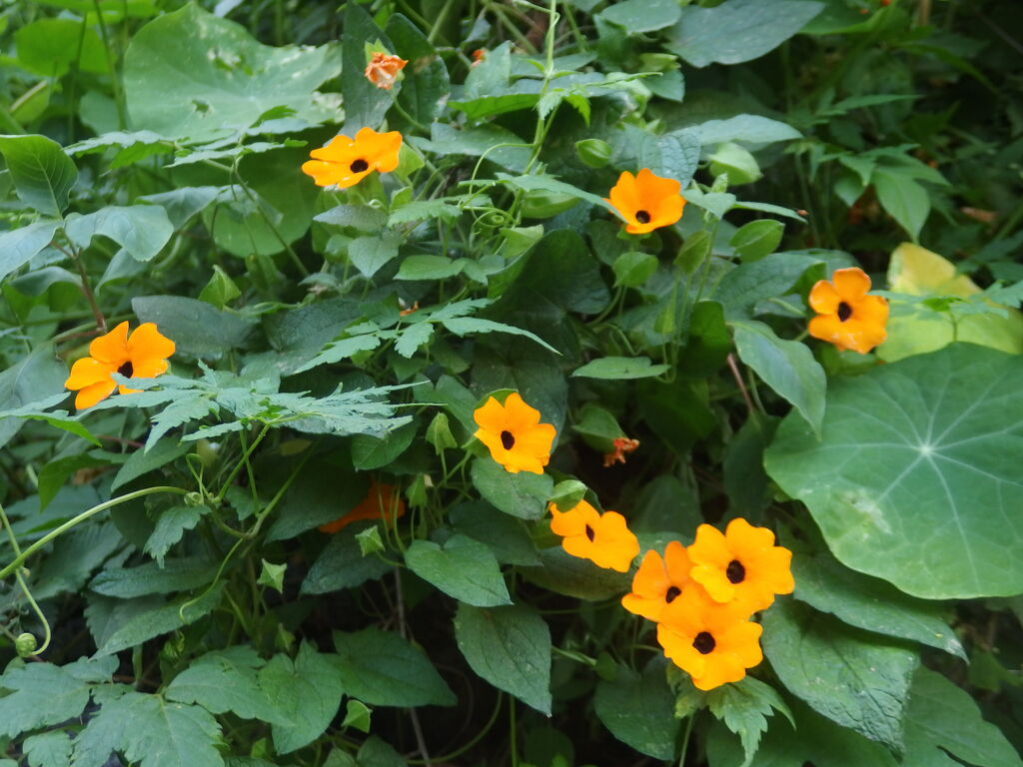
*Thunbergia alata*, San Cristóbal de La Laguna (Tejina), January 2017.

In January 2017, it was observed for the first time in Tenerife, in a ravine in an urban environment in Tejina (Fig. 10).

#### Urochloa subquadripara

(Trin.) R.D. Webster, 1987.

85DB487E-DE62-59A2-B674-0B1D20E7A10D

Urochloa subquadripara Austral. Paniceae 252. 1987.

##### Distribution

TENERIFE: Arona, Palm-Mar, Calle Estornino, plantation weed, scattered individuals, 18.12.2018, *F. Verloove* 13428 (BR). https://observation.org/observation/205286784/

##### Notes

*Urochloa subquadripara* is probably native to tropical Asia and Australia. It has been introduced into the tropics worldwide and naturalised in many parts of them, often as an undesirable weed. It was recently reported for the first time from the Canary Islands. In La Palma, the species is naturalised very locally in an irrigated lawn (Otto and Verloove 2020).

In December 2018, it was found as a weed in an ornamental plantation in Palm-Mar in Tenerife.

#### Wigandia kunthii

Choisy, 1833.

08F77B2E-A75D-5E1B-8C8E-CD3AB2E350BA

Wigandia kunthii Mém. Soc. Phys. Genève 6: 116. 1833.
Wigandia kunthii Syn. (?): *W. urens* (Ruiz & Pav.) Kunth, Nov. Gen. Sp. (quarto ed.) 3: 127. 1818 [1819].

##### Distribution

TENERIFE: Los Silos, Barranco de Las Guardias, close to TF 42, roadside, 18.11.2016, *F. Verloove* 12689 (BR). https://observation.org/observation/205287066/

##### Notes

This species, a native of the Caribbean and Central America, is sometimes grown as an ornamental, just like *Wigandia caracasana* Kunth. The latter is locally naturalised in the northern parts of Tenerife, especially near Puerto de la Cruz. It is increasing lately. *W. kunthii* was also recorded in Tenerife in 2016, apparently for the first time in the Canary Islands. A small colony, consisting of few individuals, is naturalised in the valley of Barranco de Las Guardias, in Los Silos.

*Wigandia kunthii* is much reminiscent of *W. caracasana*. Both mostly differ in the type of indumentum: the former is a shaggy-strigous plant, with pungent stinging long bristles up to 4 mm long and green lower leaf surfaces, whereas the latter has a shorter, glandular-viscid pubescence and paler lower leaf surfaces (Anonymous 2015). Although strikingly different in leaf indumentum, plants with more or less intermediate characters have been observed in Puerto de la Cruz. It is unclear whether these represent hybrids or rather indicate a weak separation between these two species.

*Wigandia kunthii* is sometimes considered conspecific with the Peruvian species *W. urens* (Ruiz & Pav.) Kunth. These two species have the characteristic stinging hairs in common, hence the specific epithet of the latter (‘urens’).

According to Anonymous (2015), *Wigandia kunthii* is the most widely naturalised species of the genus. In Italy, both are locally naturalised, like in Tenerife.

### Miscellaneous records on selected species

#### Balanites aegyptiaca

Delile, 1813

4A3DFE42-7F7D-55BA-9C7D-4A1C6D800263

Balanites aegyptiaca Descr. Egypte, Hist. Nat. 2: 221. 1813.

##### Distribution

TENERIFE: Arona (Guargacho), Calle Guayadeque, vacant lot in village, bare open ground, a single self-sown individual (not seen planted in the area), 24.06.2016, *F. Verloove* 12504 (BR). https://observation.org/observation/204907350/

##### Notes

*Balanites aegyptiaca* is a small tree, native to much of Africa and parts of the Middle East. It naturally occurs in Morocco, relatively near to the Canary Islands. It is sometimes grown for its edible fruit or, less often, as a medicinal plant.

In 2011, a single young individual of this species was found near to the port area in Los Cristianos in Tenerife (Sánchez de Lorenzo Cáceres 2012). The species was most likely introduced by African immigrants. In the intervening years, the same individual was also repeatedly seen by us.

In 2016, a further young individual of *Balanites aegyptiaca* was observed on rough ground in another village in the southern part of Tenerife, Guargacho. The introduction vector in this locality is unknown.

#### Callistemon viminalis

(Gaertn.) Cheel, 1830.

88E2E814-77F4-5A07-9B0D-6DE907827810

Callistemon viminalis Hort. Brit. 197. 1830.

##### Distribution

TENERIFE: Arona, Cho, TF-655 between Guaza and Las Chafiras, roadside, crack in concrete, a single self-sown shrub, 19.01.2019, *F. Verloove* 13436 (BR); Santa Cruz de Tenerife, Tabaiba Baja, Av. Maritima, foot of steep rock, at least five individuals, self-sown, 20.12.2019, *F. Verloove* 13737 (BR). https://observation.org/observation/205287115/; https://observation.org/observation/205287128/

##### Notes

This Australian ornamental shrub was recently reported for the first time in the wild from the Canary Islands (Gran Canaria and Tenerife) (Verloove 2017). Since then, it was repeatedly observed again, especially in Gran Canaria and a future, local naturalisation seems inevitable. *Callistemon viminalis* produces large quantities of very tiny seeds that are easily wind-dispersed. Wherever germination conditions are suitable, the species can reproduce from seed, for instance, in barrancos close to urbanisations.

In recent years, it was recorded again in two new localities in Tenerife.

#### Grevillea robusta

A. Cunn. ex R.Br., 1830

F4C6E9B3-46E2-5A30-9CA6-FC3E370C14F2

Grevillea robusta Suppl. Prodr. Fl. Nov. Holl.: 24. 1830.

##### Distribution

TENERIFE: Puerto de la Cruz, Barranco Martíanez, in the depth of the ravine, at least five self-sown, up to 300 cm tall saplings, 15.12.2019, *F. Verloove* 13734 (BR). https://observation.org/observation/205287159/

##### Notes

This Australian tree is much planted in the Canary Islands and sometimes seen self-sown in the immediate vicinity of planted individuals. It has been reported as such from Gran Canaria, La Palma and Tenerife (Kunkel 1976, Otto and Verloove 2016, Verloove 2017, Verloove et al. 2018). All these records were considered to be ephemeral.

However, in the Barranco Martíanez ravine in Puerto de la Cruz, an incipient naturalisation process was recently observed.

#### Passiflora caerulea

L., 1753

8757C6EB-7608-5E18-9211-DDA9A152139D

Passiflora caerulea Sp. Pl. 2: 959–960. 1753.

##### Distribution

TENERIFE: Santa Cruz de Tenerife, Barranco Santos close to La Ermita, dry riverbed, a single individual, 13.11.2016, *F. Verloove* 12690 (BR). https://observation.org/observation/205287176/

##### Notes

This South American species (Argentina, Brazil, Paraguay and Uruguay) is one of the most widely grown passion fruits. It easily escapes and subsequently naturalises. Although several species of this genus have been reported from the Canary Islands, *Passiflora caerulea* apparently is lacking in contemporary flora lists and databases (e.g. Acebes Ginovés et al. 2010). Its local escape, however, already was reported at the beginning of the 20^th^ century. Lindinger (1926) found it in La Laguna where it was reproducing clonally and considered to be harmful. In recent times, its presence in this area was confirmed: the species was found in the dry river bed of the Santos ravine in Santa Cruz de Tenerife in November 2016.

*Passiflora caerulea* is easily separated from the other species found as escapes in the Canary Islands, even at the vegetative stage, by its large, almost reniform stipules.

## Supplementary Material

XML Treatment for Acacia decurrens

XML Treatment for Acacia mearnsii

XML Treatment for Atriplex nummularia

XML Treatment for Bellis perennis

XML Treatment for Caesalpinia pulcherrima

XML Treatment for Chenopodium probstii

XML Treatment for Coccoloba uvifera

XML Treatment for Commelina benghalensis

XML Treatment for Cuphea hyssopifolia

XML Treatment for Ensete ventricosum

XML Treatment for Eragrostis virescens

XML Treatment for Eucalyptus camaldulensis arida

XML Treatment for Eucalyptus cladocalyx

XML Treatment for Euryops chrysanthemoides

XML Treatment for Ficus elastica

XML Treatment for Lemna minuta

XML Treatment for Lippia alba

XML Treatment for Malvastrum corchorifolium

XML Treatment for Pavonia sepioides

XML Treatment for Pittosporum tobira

XML Treatment for Plerandra elegantissima

XML Treatment for Populus × canadensis

XML Treatment for Psidium guajava

XML Treatment for Pyrostegia venusta

XML Treatment for Ruellia dipteracanthus

XML Treatment for Thunbergia alata

XML Treatment for Urochloa subquadripara

XML Treatment for Wigandia kunthii

XML Treatment for Balanites aegyptiaca

XML Treatment for Callistemon viminalis

XML Treatment for Grevillea robusta

XML Treatment for Passiflora caerulea
